# Thoracolumbar meningeal fibrosis in pugs

**DOI:** 10.1111/jvim.15716

**Published:** 2020-01-31

**Authors:** Cecilia Rohdin, Ingrid Ljungvall, Jens Häggström, Alexandra Leijon, Kerstin Lindblad‐Toh, Kaspar Matiasek, Marco Rosati, Peter Wohlsein, Karin Hultin Jäderlund

**Affiliations:** ^1^ Department of Clinical Sciences Swedish University of Agricultural Sciences Uppsala Sweden; ^2^ Anicura Albano Small Animal Hospital Danderyd Sweden; ^3^ Department of Biomedical Sciences and Veterinary Public Health (BVF), Section of Pathology Swedish University of Agricultural Sciences Uppsala Sweden; ^4^ Science for Life Laboratory, Department of Medical Biochemistry and Microbiology Uppsala University Uppsala Sweden; ^5^ Broad Institute of Harvard and Massachusetts Institute of Technology Cambridge Massachusetts; ^6^ Section of Clinical and Comparative Neuropathology, Ludwig‐Maximilians‐Universität Munich Germany; ^7^ Department of Pathology University of Veterinary Medicine Hannover Germany; ^8^ Department of Companion Animal Clinical Sciences Norwegian University of Life Sciences Oslo Norway

**Keywords:** ataxia, meninges, spinal cord

## Abstract

**Background:**

Thoracolumbar myelopathies associated with spinal cord and vertebral column lesions, with a similar clinical phenotype, but different underlying etiologies, occur in pugs.

**Objectives:**

To further characterize the clinical and neuropathological characteristics of pugs with longstanding thoracolumbar myelopathy.

**Animals:**

Thirty client‐owned pure‐bred pugs with a history of more than a month of ataxia and paresis of the pelvic limbs, suggesting a myelopathy localized to the thoracolumbar spinal cord, were included in the study.

**Methods:**

Prospective clinicopathological study. Included pugs underwent a complete neurological examination and gross and histopathologic postmortem studies with focus on the spinal cord. Computed tomography (n = 18), magnetic resonance imaging (n = 17), and cerebrospinal fluid analysis (n = 27) were performed before or immediately after death.

**Results:**

Twenty male and 10 female pugs had a median age at clinical onset of 84 months (interquartile range, 66‐96). Affected pugs presented with a progressive clinical course and 80% were incontinent. There was circumferential meningeal fibrosis with concomitant focal, malacic, destruction of the neuroparenchyma in the thoracolumbar spinal cord in 24/30 pugs. Vertebral lesions accompanied the focal spinal cord lesion, and there was lympho‐histiocytic inflammation associated or not to the parenchymal lesion in 43% of the pugs.

**Conclusions and Clinical Importance:**

Meningeal fibrosis with associated focal spinal cord destruction and neighboring vertebral column lesions were common findings in pugs with long‐standing thoracolumbar myelopathy.

**Abbreviations**
CAP: caudal articulate process
CNS: central nervous system
CSF: cerebrospinal fluid
CT: computed tomography
CVM: congenital vertebral malformation
GFAP: glial fibrillary acidic protein
IVDH: intervertebral disc herniation
MRI: magnetic resonance imaging
SAD: subarachnoid diverticula

## 1 INTRODUCTION

Myelopathy involving the thoracolumbar spinal cord is well recognized in dogs and is clinically characterized by ataxia, paresis, or paralysis of the pelvic limbs, whereas the thoracic limbs are spared.^1^ The clinical presentation in dogs with thoracolumbar myelopathy is highly variable regarding clinical onset, course, and presence of spinal pain, depending partly on the underlying etiology.^2^ In pugs however, thoracolumbar myelopathy with uniform clinical phenotype and clinical course, but of different etiologies, including spinal cord as well as vertebral column lesions, is described.^3^, ^4^, ^5^, ^6^, ^7^, ^8^, ^9^, ^10^, ^11^, ^12^, ^13^, ^14^, ^15^, ^16^, ^17^, ^18^, ^19^, ^20^, ^21^, ^22^, ^23^, ^24^, ^25^, ^26^, ^27^ Spinal cord lesions, including subarachnoid diverticula (SAD) and constrictive myelopathy, have been given attention in pugs as separate diagnoses underlying myelopathy.^11^, ^12^ The histopathological features of the affected spinal cord tissues in pugs with myelopathy have been sparsely studied, mostly limited to meningeal material removed at surgery. In that respect, both SAD and constrictive myelopathy are associated with proliferation and thickening of the meninges.^3^, ^5^, ^11^, ^12^, ^17^ Vertebral column lesions, such as congenital vertebral malformations (CVMs), including hemivertebra and hypo‐ or aplasia of the caudal articulate processes (CAPs),^4^, ^11^, ^21^, ^27^ and acquired intervertebral disc herniation (IVDH),^8^, ^13^ are inconsistently described in pugs presenting as thoracolumbar myelopathy.

The high prevalence of neurological gait abnormalities in pugs^6^, ^28^ and the growing evidence of thoracolumbar myelopathy with a uniform clinical presentation in the breed, albeit of different etiologies, indicate that further investigations and characterization are warranted in this group of dogs. Objectives of this prospective study were to further characterize the clinical condition of pugs with longstanding thoracolumbar myelopathy, and through postmortem studies describe their neuropathological characteristics.

## 2 MATERIALS AND METHODS

The study was approved by the local Ethical Committee (Animal Ethics Committee of Sweden [Uppsala Djurförsöksetiska Nämnd] C202/2014b). Client‐owned pure‐bred Swedish pugs were included in the study between 2011 and 2018 at Albano Animal Hospital, Danderyd, and at the University Animal Teaching Hospital, Uppsala.

### 2.1 Inclusion criteria

Pugs were included in the study provided that they fulfilled the following criteria: a history of more than 1 month of a bilateral pelvic limb gait abnormality described by the owner as incoordination and weakness (onset of clinical signs was determined from the owner's first recognition of incoordination and weakness in the pelvic limbs), and a neurological examination confirming pelvic limb ataxia and paraparesis suggestive of a myelopathy localized to the thoracolumbar spinal cord. For the dog to be included, the owners also agreed to donate their dog for postmortem studies after naturally occurring death or euthanasia.

### 2.2 Exclusion criteria

Dogs were excluded if histopathology of the thoracolumbar spinal cord was not performed at the time the pug died or was euthanized.

### 2.3 Study population

Signalment and clinical variables were included in the data set. A thorough case history, including onset and course of clinical signs, presence of incontinence (fecal/urinary), signs indicating the dog was in pain, and result of any given treatment, was obtained at the time of inclusion. All pugs underwent a complete neurological examination performed the same day they were euthanized, by a board‐certified neurologist (C. Rohdin). After euthanasia, the included pugs underwent a full postmortem examination with focus on the spinal cord and vertebral column. If practically possible, the included pugs underwent advanced diagnostic imaging, including magnetic resonance imaging (MRI) and computed tomography (CT), as well as cerebrospinal fluid (CSF) sampling and analysis, and repeat neurological examinations (by C. Rohdin). Advanced imaging was carried out before or immediately after death in order to associate any potential spinal cord lesion with the presence of vertebral column lesions, as well as to guide the histopathologic investigation of the spinal cord. Some of the recruited dogs were also included in another study investigating the presence of CVMs in pugs with and without neurological deficits.^18^

### 2.4 Imaging studies

Computed tomography was performed with 2 mm slice thickness, using a dual slice CT scanner (Siemens, Somatom Spirit, Siemens Munich, Germany), or a 64 slice CT scanner (Siemens, Somatom Definition, Siemens Munich, Germany). The studies were reconstructed with a soft tissue and a bone algorithm using medium and high‐frequency reconstruction algorithms. For CT studies, the examination included the entire thoracic and lumbar vertebral column and the presence of vertebral malformations, such as hemivertebra, associated kyphosis, transitional vertebrae, and hypo‐ or aplasia of the CAPs was evaluated.

The MRI studies were acquired using either a magnet operating at 0.2 Tesla (Esaote Vet‐MR Grande, Esaote, Genoa, Italy), or at 1.5 Tesla (Siemens, Magnetom Essenza, Siemens Munich, Germany). At a minimum, the MRI study included sagittal T1‐ and T2‐weighted images of the entire thoracolumbar spine and transverse T1‐ and T2‐weighted images of the area of interest. Intervertebral discs were described as compressing (mildly, moderately, severely), or not compressing the spinal cord.

All CT and MRI studies were submitted to and evaluated by a single board‐certified imager at a diagnostic imaging company (VetImaging of New York, New York).

### 2.5 Cerebrospinal fluid analysis

The CSF samples were taken at the University Animal Teaching Hospital, Uppsala, Sweden, or at Albano Animal Hospital, Danderyd, Sweden, and analyzed within 30 minutes of collection. The analyses included total leucocyte‐ and erythrocyte cell counts, and differential leucocyte counts, and measurement of protein concentration (turbidimetry). Protein concentration was considered to be increased if >0.25 g/L at the cerebellomedullary cistern, or >0.40 g/L at the lumbar cistern.^29^ Cerebrospinal fluid was interpreted to have pleocytosis if there were >5 nucleated cells/μL, and pleocytosis was classified by the primary cell population if 1 cell type comprised >50%.^29^

### 2.6 Pathology

Gross pathology was performed at the Section of Pathology, Swedish University of Agricultural Sciences (site 1) or at the National Veterinary Institute, Uppsala, Sweden (site 2).

The entire central nervous system (CNS) were carefully sampled and fixed in 10% neutral buffered formalin for transport to a board‐certified pathologist. After the spinal cord had been evacuated, the vertebral column was carefully examined and cut sagittally. Midsagittal sections were performed of thoracolumbar vertebrae and intervertebral discs with macroscopically degenerative changes, and overt disc herniations on macromorphology, or where compression of the spinal cord had been demonstrated on MRI. Discs adjacent to disc herniations were also included. The sections were placed in 10% neutral buffered formalin and decalcified using 20% formic acid (Kristensens solution).

Histopathological examination of the CNS was performed at site 2, at the Department of Pathology, University of Veterinary Medicine, Hannover (site 3), or at the Section of Clinical and Comparative Neuropathology, Ludwig‐Maximilians‐Universität, Munich (site 4), Germany. After fixation, specimens of various anatomical sites of the brain and spinal cord were processed for routine histopathology, embedded in paraffin wax and sectioned at 3‐4 μm. Tissues were stained using routine hematoxylin and eosin (HE), and examined by light microscopy.

Histopathology of the vertebral column was performed at site 1. Decalcified tissue was embedded using paraffin wax and processed to 4‐5 μm sections. Tissues were routinely stained with HE. The overall morphology and integrity of the intervertebral discs and adjacent vertebrae, in addition to other apparent features, were assessed and recorded.

### 2.7 Statistics

Descriptive statistical analyses were performed using a commercially available statistical software program (JMP Pro v. 11.2.0, Cary, North Carolina). Continuous variables are presented as means and SDs and as medians and interquartile range (IQR). Associations between clinical variables and pathological findings were investigated using the Chi‐square, Fishers exact test. Level of statistical significance was set at *P* < .05.

## 3 RESULTS

### 3.1 Study population characteristics

Thirty pugs fulfilled the inclusion criteria with pathology being performed by the time the pug was euthanized. Information about dog signalment, clinical variables, CSF findings, and whether or not the dog was receiving medical treatment is presented in Table 1. No pug had undergone neurosurgical intervention.

**Table 1:** Distribution of signalment, clinical variables, cerebrospinal fluid, and histopathological findings of lympho‐histiocytic inflammation in the central nervous system of the 30 pugs of the present study. Data on signalment and incontinence is compared to pugs in the general Swedish pug population^a^

Variable	The 30 pugs of the present study	The general Swedish pug population^a^
Number of pugs	30	550
Sex		
Male	18 (60.0%)	38.7%
Male (neutered)	2 (6.7%)	9.1%
Female	10 (33.3%)	43.1%
Female (spayed)	0 (0)	9.1%
Median weight (kg)	9.0 (IQR, 8.4‐10.0)	9 (IQR, 8‐10)
Coat color		
Black	5 (16.7%)	24.6%
Fawn	25 (83.3%)	71.0%
Median age (months) at clinical onset	84 (IQR, 66‐96)	‐
Range of age at clinical onset (months)	7‐124	
Incontinence	24 (80.0%)	
Fecal	24 (80.0%)	3.6%
Urinary	7 (23.3%)	6.7%
Presented with pelvic limb gait abnormality before the onset of incontinence	23 (95.8%)	
Urinary incontinence developed before fecal incontinence	1 (4.2%)	
Abnormal CSF	19/27 (70.4%)	‐
Mononuclear pleocytosis	14/27 (51.9%)	
Increase in total protein	17/27 (63.0%)	
Mean duration of clinical signs (months) before euthanasia:		‐
In all Pugs	12.7 (SD, 7.9)	
In pugs with lympho‐histiocytic inflammation in the CNS on histopathology^b^	9.8 (SD, 7.5)	
In pugs (n = 2) *with* lympho‐histiocytic leptomeningitis	2.8 (SD, 1.8)	
In pugs (n = 28) *without* confirmed lympho‐histiocytic leptomeningitis	13.6 (SD, 7.7)	
Pugs receiving ongoing medical treatment	7/30 (23.3%)	
Corticosteroids	4/7	
NSAIDs	2/7	
Corticosteroids + cyclosporine	1/7	
Mean duration of clinical signs (months) before euthanasia		
In pugs receiving treatment	11.9 (SD, 5.8)	
In pugs not receiving treatment	12.9 (SD, 8.5)	

Abbreviations: CSF, cerebrospinal fluid; IQR, interquartile range; NSAIDs, non‐steroidal anti‐inflammatory drugs.

a From Reference 28.

b Four out of the 13 pugs were receiving treatment with an anti‐inflammatory agent at the time of sampling.

All 30 pug owners described an insidious, progressive course of their pug's pelvic limb gait abnormality. Mean duration of clinical signs before veterinary consultation was 7.1 months (SD, 6.9). None of the owners perceived their dogs to be in pain, but a reluctance to go for walks was reported by the owners of 13 pugs.

At the physical examination, all 30 pugs presented with moderate to severe pelvic limb muscle atrophy. Spinal pain was not recognized in any of the pugs upon examination. In 13 pugs, a repeated neurological examination had been performed. In 3 of them, the pelvic limb flexor reflexes gradually became weaker, with loss of muscle tone in the pelvic limbs, at reexaminations performed within 9.5, 11, and 12 months after the initial examination. These 3 pugs were examined neurologically on 7, 4, and 3 occasions respectively. In 2 out of these 3 pugs, with a normal perineal reflex upon initial examination, loss of the perineal reflex and anal tone developed over time.

All 30 pugs were still ambulatory at the day of euthanasia. Twenty‐eight pugs were euthanized because of their gait abnormality or because of their associated incontinence. Other causes for euthanasia (n = 2) were pyometra (n = 1) and osteosarcoma of the mandibula (n = 1). The pug with pyometra had a neurological examination performed, confirming pelvic limb ataxia and paraparesis, 1 month before acute onset of clinical signs of the pyometra. The pug with osteosarcoma had a neurological examination, confirming pelvic limb ataxia and paraparesis, and a CT of the entire thoracolumbar vertebral column, with no signs of vertebral involvement, performed 2 months before euthanasia.

### 3.2 Imaging studies

**Figure 1:**
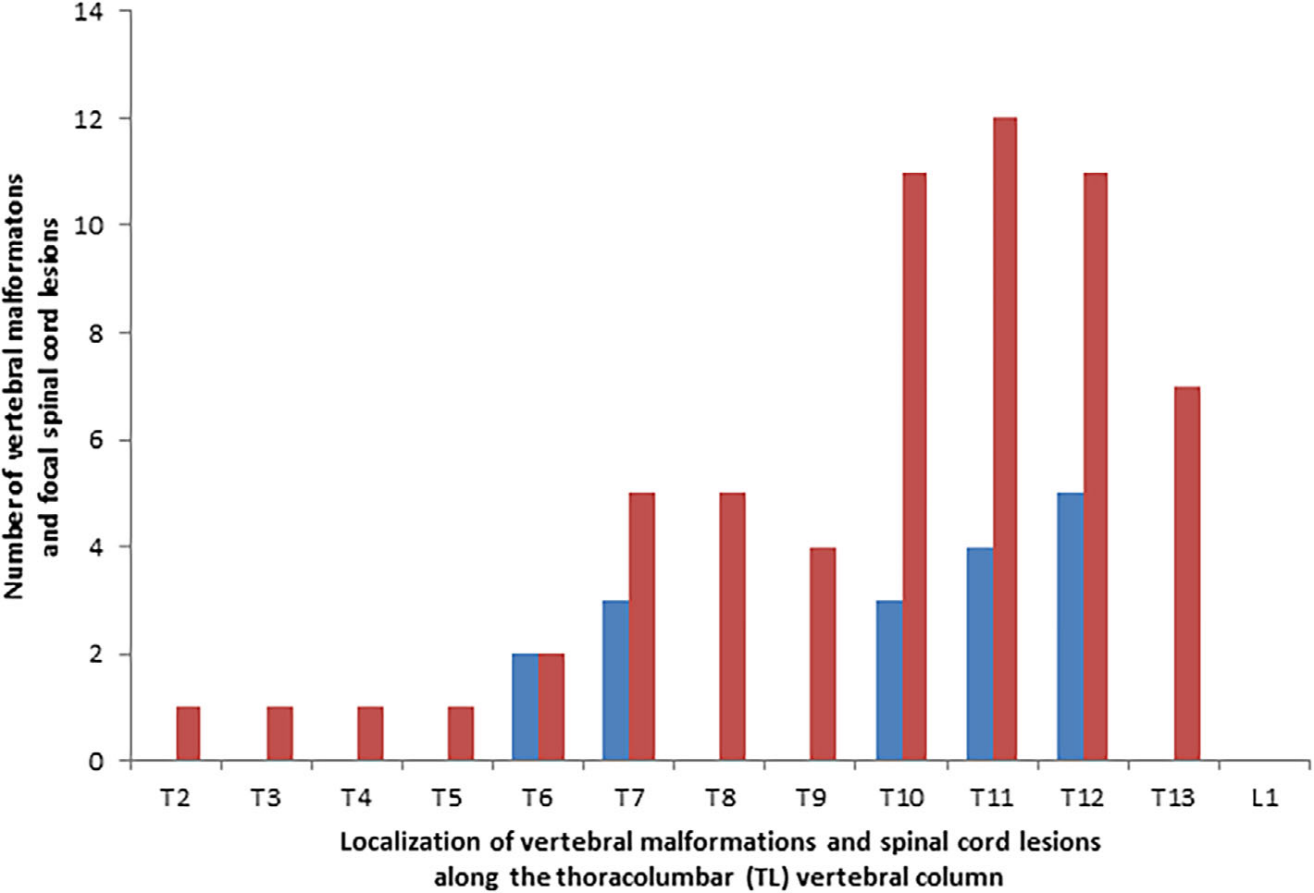
Distribution of the focal thoracolumbar spinal cord lesions and their adjacent vertebral malformations, diagnosed by computed tomography, in 17 pugs with long‐standing thoracolumbar myelopathy. Vertebral malformations included hemivertebra, hypo‐ or aplasia of the caudal articular process, and thoracolumbar transitional vertebrae. Blue bars represent focal spinal cord lesions. Red bars represent vertebral malformations

**Figure 2:**
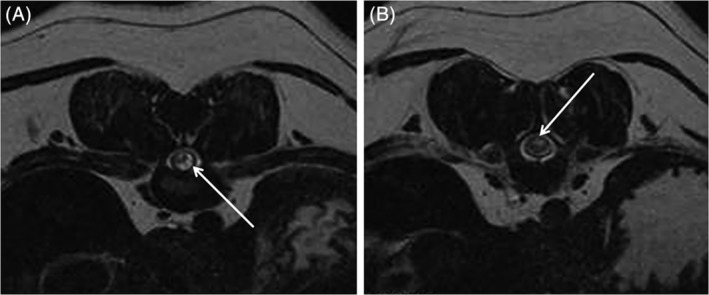
Transversal T2W spinal cord magnetic resonance images at the level of T10‐T11 in a pug. A ventrolateral subarachnoid diverticula (SAD) (A) is located immediately cranial to the intramedullary T2W hyperintensity (B). White arrow points at SAD in (A), at T2W hyperintensity in (B)

Fifteen pugs underwent both CT and MRI imaging. All CT studies (n = 18) were performed immediately after death. Magnetic resonance imaging was performed under general anesthesia (n = 7) or immediately after death (n = 10). Mean time between MRI and euthanasia in the 7 pugs examined under anesthesia was 6.3 months (SD, 4.2 months). In the 18 pugs examined by CT, 64 CVMs were identified, mean 3.6 (SD, 2.0), in the thoracolumbar vertebral column. In CT‐examined pugs with histopathologically confirmed focal spinal cord lesions (n = 17), at least 1 CVM presented immediately adjacent to the focal lesion in 16 pugs (94%) (Figure 1). Four hemivertebra and 12 hypo‐ or aplasia of the CAPs were found accompanying the focal lesion. In addition, 3 pugs presented transitional thoracolumbar vertebrae adjacent to the focal spinal cord lesion. In the remaining pug examined by CT, a herniated intervertebral disc, diagnosed by MRI, was located immediately adjacent to the focal spinal cord lesion. In all but 1 pug (n = 16), examined by MRI (n = 17), the spinal cord lesion presented with a focal intramedullary T2W hyperintensity. The 17th pug presented with a more extensive spinal cord T2W hyperintensity (T10‐L1). A SAD, located immediately cranially or caudally to the focal intramedullary T2W hyperintensity, was diagnosed by MRI in 9/16 (56%) pugs (Figure 2). An IVDH, compressing the spinal cord at the intramedullary T2W hyperintensity, was diagnosed by MRI in 5 pugs. The disc compression was considered mild in 2 and moderate in 3 pugs.

### 3.3 Analysis of CSF

Cerebrospinal fluid was sampled from the cisterna magna (n = 26) or from the lumbar area (n = 1) when the dogs were under general anesthesia (n = 1) or in association with euthanasia (n = 26). The median number of mononuclear cells in the CSF was 6/μL (IQR, 2‐18) (range, 0‐217); of protein 0.3 g/L (IQR, 0.25‐0.42) (range, 0.14‐0.56); and of erythrocytes 5/μL (IQR, 0‐16) (range, 0‐445) (Table 1). The CSF of the pug with pyometra was unremarkable. The CSF of the pug with osteosarcoma showed an elevated total protein concentration with normal cell count.

### 3.4 Pathology

Gross pathology was performed at site 1 (n = 29), and at site 2 (n = 1).

Histopathology of the CNS was performed at site 3 (n = 19), at site 4 (n = 10), and at site 2 (n = 1).

Fifteen dogs had intervertebral discs evaluated histopathologically (site 1).

#### 3.4.1 Macroscopic findings

Vertebral column pathology included hemivertebra (n = 6), with (n = 4) or without (n = 2) kyphosis, and herniating intervertebral discs (n = 15). The morphology of the CAPs could not be assessed, as they were destroyed by the processing procedure. There was occasional focal spinal cord atrophy of the thoracolumbar spinal cord.

#### 3.4.2 Histopathology of the CNS

Lesions were confirmed in the CNS of all examined pugs including meninges, spinal cord, and nerve roots. Meningeal fibrosis and concomitant spinal cord parenchymal lesions were a consistent finding in 29/30 pugs.

**Figure 3:**
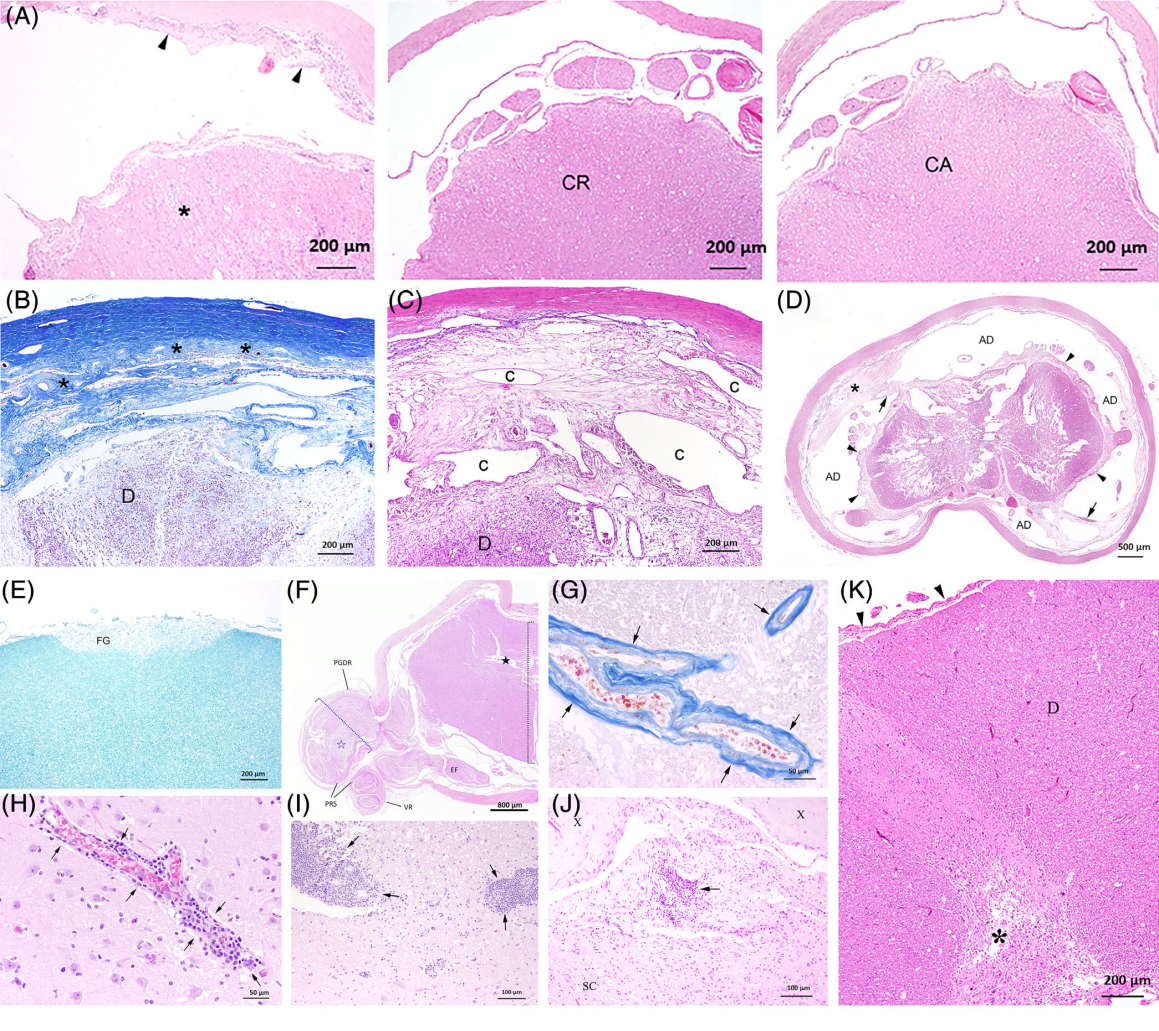
Histopathologic findings in the spinal cord from pugs affected by thoracolumbar myelopathy, showing: A, focal spinal cord lesion with segmental subdural fibrosis and adjacent slices without the fibrosis only a few millimeter cranially (CR) and caudally (CA) from the damaged area along the spinal cord; HE staining. B, Severe meningeal fibrosis with adhesion of lepto‐ and pachymeninges (asterisks). “D” indicates dorsal tract; Azan staining. C, Formation of diverticula with multiple compartments “C” of the subarachnoid space. “D” indicates dorsal tract; HE staining. D, Focal malacia with almost total destruction of the spinal cord, leptomeningeal (arrowheads) and subdural (asterisk) fibrosis with focal adhesions (arrow) and numerous arachnoidal diverticula (AD). HE staining. E, Myelin loss in the fasciculus gracilis (FG), of the dorsal funiculus, at the level of the cervical spinal cord; Luxol fast blue staining. F, Compression radiculopathy at T7. Note the enlarged transsectional diameter (blue dotted bracket) of preganglionic dorsal root (PGDR) in relation to the maximal dorsoventral diameter of the respective spinal cord segment (black dotted bracket). The most affected dorsal root further shows endoneurial oedema (blue‐lined asterisk). Both extradural parts of PGDR and ventral root (VR) present with thickening of the periradicular sheath (PRS), whereas intradural ventral rootlets are affected by a focal endoneurial fibrosis (EF), HE staining. G, Perivascular fibrosis (arrows) at the level of L3, spinal cord; Azan staining. H, Mild perivascular lympho‐histiocytic inflammation (arrows) in the cerebrum; HE staining. I, Moderate lympho‐histiocytic inflammation (arrows) in the spinal cord; HE staining. J, Moderate lympho‐histiocytic leptomeningitis (arrows) adjacent to a thoracic, malacic, focal spinal cord (SC) lesion. “X” represent pachymeninges; HE staining. K, Lack of leptomeningeal fibrosis (arrowheads) adjacent to a focal malacic cervical spinal cord lesion (*) in the dorsal horn. “D” represents dorsal tract; HE staining

Meningeal fibrosis was confirmed in the thoracolumbar spinal cord of 29 pugs. Focal, circumferential, meningeal fibrosis, limited in length along the spinal cord, in many cases involving only a few millimeters (Figure 3A), was present in 28 pugs—27 in the T5‐L1 region and 1 at L5/L6. The focal meningeal proliferation was profound in 27 pugs (Figure 3B), and mild in 1 pug. In 1 other pug, the meningeal fibrosis extended the length of the spinal cord between T3 and T13. The meningeal fibrosis involved the pachy‐ and leptomeninges in 13 (n = 13); pachymeninges in 3 (n = 3); leptomeninges in 3 (n = 3); and involved the meninges without distinction in 9 (n = 9) dogs. Focal leptomeningeal proliferation and adhesions were in 9 pugs associated with formation of a diverticula, in some creating multiple compartments of the subarachnoid space (Figure 3C).

Mild to moderate meningeal proliferation was also recognized throughout the CNS unrelated to the focal spinal cord pathology in 14 pugs.

A single, thoracolumbar, focal spinal cord lesion was repeatedly found accompanying the meningeal fibrosis in 28 pugs. In 1 other pug, the lesion was more extensive, presenting between T10 and L1. The parenchymal spinal cord lesions were characterized by malacia (n = 24), by syringomyelia (n = 4), or by hydromyelia (n = 1). The level of these focal lesions corresponded to the intramedullary T2W hyperintensity on MRI. The focal malacic lesions within the spinal cord were delineated by microgliosis, astrogliosis, gemistocytes, and astrocytosis, forming astroglial scars (Figure 3D). The malacic lesions, with almost total destruction of the parenchyma, were clearly demarcated from adjacent spinal cord cranially and caudally (Figure 3A). Clinical variables and histopathological findings between pugs with focal parenchymal spinal cord pathology characterized by malacia and the group of pugs with syringo/hydromyelia are presented in Table 2.

**Table 2:** Distribution of clinical variables and histopathological findings of lympho‐histiocytic inflammation in the central nervous system of 29 pugs with spinal cord lesions confirmed by histopathology. Level of statistical significance was set at *P* < .05

Variable	Spinal cord lesion represented by malacia (n = 24)	Spinal cord lesion represented by syringomyelia/hydromyelia (n = 5)	*P* value
Median age (months) at clinical onset	84 (IQR, 67.5‐96.0)	68 (IQR, 54.5‐108.5)	.97
Mean duration of clinical signs (months) before euthanasia	13.9 (SD, 7.8)	7.1 (SD, 7.4)	.06
Incontinence			
Fecal	20/24 (83.3%)	2/5 (40.0%)	.27
Urinary	7/24 (29.2%)	0/5	.08
Pugs with lympho‐histiocytic inflammation in the CNS on histopathology	8/24 (33.3%)	4/5 (80.0%)	.05

Abbreviations: CNS, central nervous system; IQR, interquartile range.

Wallerian‐like degeneration could be traced further cranial from the malacic spinal cord lesions, mainly through the fasciculus gracilis in the cervical spinal cord (Figure 3E). Compared to the, in general, severe focal thoracolumbar spinal cord lesions, these neurodegenerative lesions were mild.

Nerve root entrapment and vascular encasement was found with meningeal vessels and nerve roots embedded in the circumferential meningeal fibrosis. Loss of nerve fiber density, seen involving both dorsal and ventral nerve roots (Figure 3F), hypertrophy of the dentate ligaments, and intradural tethering was found including the thoracolumbar but also the cervical and lumbosacral spine. In addition, perivascular fibrosis with collagen deposits were found throughout the CNS (Figure 3G), involving areas with and without meningeal proliferation.

Varying degrees of lympho‐histiocytic CNS inflammation, including encephalitis (n = 9) (Figure 3H), myelitis (n = 8) (Figure 3I), and pachymeningitis with plaque‐like mineralizations (n = 2), were found in 13 pugs. Mild to moderate lympho‐histiocytic leptomeningitis was found adjacent to the focal spinal cord destruction in 2 pugs (Figure 3J). The pug euthanized because of pyometra had perivascular cuffs of lympho‐histiocytic inflammatory cells in the brain. Evidence of lympho‐histiocytic infiltration of the CNS was not confirmed in the pug with osteosarcoma.

Severe gray matter destruction was recognized in the cranial cervical spine in 3 pugs. One of these pugs had had surgery to remove a SAD in the dorsal C2‐C3 spinal cord area 4 years before presenting with signs of a T3‐L3 myelopathy. These lesions displayed the same features as the lesions in the thoracolumbar spinal cord, with (n = 2) or without (n = 1) (Figure 3K) associated meningeal fibrosis. There was no evidence of fibrocartilaginous material in the vasculature supplying the affected cervical or the thoracolumbar spinal cord tissue.

Meningeal fibrosis with a concomitant spinal cord lesion was absent in 1 pug. At 10 months before euthanasia, this pug was diagnosed by MRI (1.5 Tesla) with a focal intramedullary T2W hyperintensity and a probable SAD at T6/7. Histopathology displayed neurodegenerative features with mild‐severe dilated myelin sheaths, swollen axons, and myelinophagia in the cervical, thoracic, and lumbar spinal cord, however, no focal lesions was identified.

#### 3.4.3 Histopathology of the vertebral column

Fifteen out of 30 pugs had a total of 39 thoracolumbar discs evaluated histopathologically. Herniating intervertebral discs were found at the identical site as the focal lesion in 4 out of 15 pugs. On MRI, these 4 herniations were mildly moderately compressing the spinal cord. In 1 pug with evidence of moderate spinal cord compression from a disc diagnosed on MRI, IVDH could not be confirmed by histopathology 8 months later.

**Figure 4:**
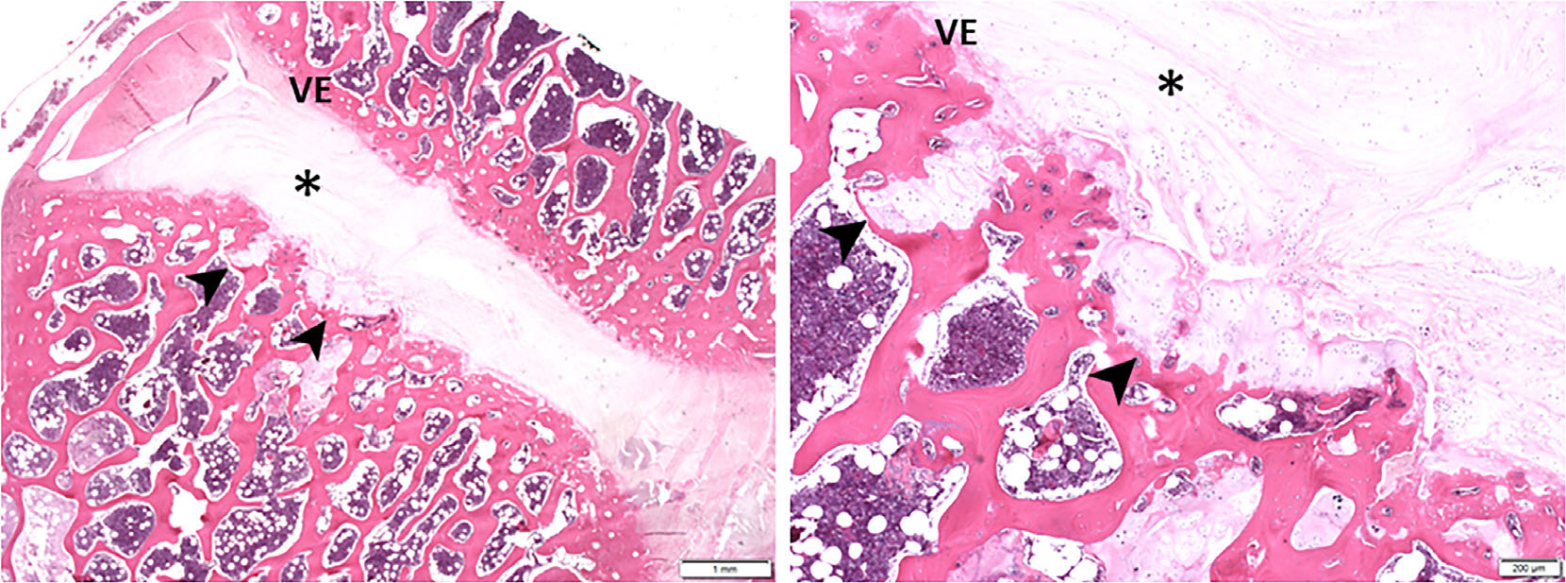
Protrusion of chondroid tissue of the nucleus pulposus into the adjacent endplate, a so‐called Schmorl's node (arrowheads), in a pug. VE, vertebral endplate; *, intervertebral disc; HE staining

In 6 of the 15 examined pugs, protrusions of chondroid tissue, presumably originating from the nucleus pulposus with chondroid metaplasia, into the adjacent endplate and subsequently into the subchondral bone, so called Schmorl's nodes, were found (Figure 4). Schmorl's nodes were found in vertebrae adjacent to, but also unrelated to, the focal lesion in the spinal cord.

## 4 DISCUSSION

Apart from sharing a uniform clinical presentation and course of the disease, pugs in this study also exhibited similar neuropathological features, including thoracolumbar meningeal fibrosis and concomitant spinal cord destruction. Although the homogenous clinical and pathological findings could represent a true disease, the additional finding of meningeal fibrosis unrelated to the thoracolumbar spinal cord lesion more likely suggest a predisposition, possibly breed related, to meningeal proliferation. The profound meningeal fibrosis could interfere with vascular supply to the spinal cord as well as cause obstruction of CSF flow with subsequent parenchymal spinal cord destruction.

Although the finding of concomitant CNS inflammation and neighboring vertebral column pathology might be unrelated findings, their high, and for CNS inflammation unexpected, prevalence suggest they are part of the underlying etiology.

Histopathological evidence of ongoing mild to severe lympho‐histiocytic CNS inflammation, presenting distant to or at the focal spinal cord lesion, was found in 43% of the examined pugs. In addition, 52% pugs presented with a cell composition in the CSF indicating, in general mild, intrathecal mononuclear inflammation. Although an infectious etiology was considered unlikely because of the stereotype and longstanding history, this cannot be entirely excluded. On the other hand, glial fibrillary acidic protein (GFAP), the hallmark intermediate filament protein in astrocytes,^30^ has the potential to induce an immune‐mediated reaction in both dogs and humans.^31^, ^32^, ^33^, ^34^ Leakage of GFAP has been shown in clinically normal pugs and was suggested to be the result of a breed‐specific astrocyte fragility.^32^, ^35^ Astrocyte activation, in response to a spinal cord insult from, for example, an IVDH, and/or by appropriate upregulation of GFAP,^36^, ^37^ may have triggered an inflammatory, possibly autoimmune, response in the CNS contributing to the lesions seen in the pugs of this study.^38^

The spinal cord lesions from the pugs with thoracolumbar myelopathy in this study shared many features with the spinal cord of laboratory dogs, used in research about adhesive, also called constrictive, arachnoiditis.^39^, ^40^ Adhesive arachnoiditis, a rare chronic myelopathy in people, is characterized by meningeal fibrosis that has been described to cause disruption of the vascular supply and the development of arachnoid diverticula, syringomyelia and myelomalacia.^41^, ^42^, ^43^, ^44^, ^45^, ^46^ Familiar adhesive arachnoiditis, with a suggested genetic influence, has been described.^47^, ^48^, ^49^ In the present study, active inflammation involving the arachnoidea at the focal spinal cord lesion was found in only 2 of the affected pugs. These 2 pugs were euthanized early after the onset of clinical signs (1.5 and 4 months), which brings to question if a focal arachnoiditis was no longer present in the other affected pugs by the time they were euthanized.^3^, ^50^ This was, however, not unequivocally supported by the overlap in range of duration of clinical signs before euthanasia in pugs *without* (1.5‐30 months) and those *with* inflammatory infiltration of the arachnoidea. Mild inflammatory meningeal changes have previously been described in pugs with SAD.^3^, ^5^, ^51^

Pugs with intraparenchymal malacia presented with a longer duration of clinical signs before euthanasia and less often with lympho‐histiocytic inflammation in the CNS on histopathology compared to pugs with syringo/hydromyelia. Although not statistically significant, this may indicate that there is an early inflammatory phase and a later chronic proliferative state in which fibrosis and adhesions become permanent in pugs with a longstanding thoracolumbar myelopathy, as suggested in human literature related to adhesive arachnoiditis.^52^

Vertebral column lesions, including CVMs and IVDHs, accompanied the focal meningeal fibrosis and spinal cord lesions in pugs diagnosed by CT. Unfortunately, all pugs in the present study were not examined by advanced imaging, and some CVMs, in particular articular process dysplasias, are difficult to document without CT.^3^ Although a previous study, including pugs of similar age as in the present study, could not confirm an association between CVMs and neurological signs, it has been suggested that some CVMs in pugs are indeed clinically significant.^10^, ^11^, ^18^ Vertebral malformations, congenital or potentially acquired,^53^ may inflict direct spinal cord trauma by chronic micromotion as a consequence of vertebral canal stenosis or instability, or indirect trauma by negatively interfering with its vascular supply and thereby limiting its resistance to injury.^4^, ^7^, ^11^, ^14^, ^54^, ^55^, ^56^ Spinal cord dynamic compression occurs in pugs with SAD and repetitive spinal cord injury could be a potential cause of thoracolumbar SADs.^25^ Future biomechanical studies are required to confirm a potential association between a greater risk to develop neurological signs in pugs and the nature and spatial distribution of their CAP malformations.^7^, ^10^

An interesting finding was the prolapse of the nucleus pulposus through the cartilaginous endplate into the body of the vertebral body, consistent with a Schmorl's node. Schmorl's nodes are uncommonly described endplate abnormality in canines.^57^, ^58^, ^59^ There are different hypotheses for the pathogenetic background of the Schmorl's nodes, including increased axial load and disc degeneration.^60^ An association with ischemic necrosis, caused by interruption of the vascular supply to the vertebral endplate, has also been proposed.^60^, ^61^ In contrast to some other vertebral column changes found in this study, the presence of Schmorl's nodes was not correlated to spinal cord lesions.

Histopathology showed intradural tethering and hypertrophy of the dentate ligaments in pugs of this study. The spinal cord, meninges, and nerve roots move within the vertebral canal upon posturing of the head and spine.^62^, ^63^ Respiration, especially forced, also has an impact on spinal cord movement.^64^ Intradural tethering and/or hypertrophy of the dentate ligament may be the consequence of increased spinal cord movement within the vertebral canal. Spinal cord movement above and below a segment of the cord where meningeal fibrosis holds the spinal cord fixed, could sustain, and potentially also contribute to progression of the injury by constant repetitive traction.^39^

Although all pugs included in this study presented with the similar, predictive, clinical phenotype, thoracolumbar meningeal fibrosis and parenchymal spinal cord pathology were not confirmed in 1 dog, which indeed had a T2W hyperintensity in the thoracolumbar spinal cord on MRI. As the extent of the parenchymal spinal cord pathology in general was limited in length along the spinal cord, it might therefore easily have been missed on trimming procedures.

The majority of pugs presented with incontinence, and fecal incontinence was more likely to develop before urinary incontinence. Reports on fecal and urinary incontinence in dogs, including pugs, with upper motor neuron spinal cord lesions have been sparse and mostly associated with SAD.^9^, ^16^, ^20^ The character of the perineal reflex of a few affected pugs changed over time and became weaker leaving the sphincter open. This suggests that the neurological dysfunction responsible for incontinence in affected pugs could be more complex than previously proposed,^20^ involving also lower motor neurons. In addition, repeated neurological examinations showed that, with time, the spinal reflexes in the pelvic limbs of some pugs became weaker with loss of muscle tone. The recognition of decreased spinal cord reflexes, including the perineal reflex, could be related to the observed nerve root pathology and needs further investigation.

Spinal pain was not described by any of the owners nor recognized upon examination in any of the pugs. However, reluctance to go for walks was described by more than a third of the owners. Although vascular compromise to the spinal cord in dogs, except initially, is described as non‐painful,^65^, ^66^ people with ischemic spinal cord lesions describe chronic, intense pain that increase with exertion.^67^ It needs to be questioned whether the decreased activity in some pugs with thoracolumbar myelopathy was because of the inability to walk normally, because of any concomitant breed related dyspnea, or, in fact, a reflection of pain.^28^, ^68^

We acknowledged that this descriptive study, having more than 1 pathologist examining the pugs, was limited by the lack of possibility for objective comparison and statistical analysis. However, the pathologists independently confirmed the major pathological findings of this study, that is, meningeal fibrosis, and concomitant spinal cord lesions, and also the presence of lympho‐histiocytic CNS inflammation. Furthermore, each of them provided valuable input related to additional pathology and potential pathogenesis of pugs with longstanding thoracolumbar meningeal fibrosis. Owner attitude and financial limitations early in the project might have resulted in exclusion of some dogs undergoing the same diagnostic protocol and may have limited the opportunity to evaluate the importance of neighboring vertebral lesions.

## 5 CONCLUSIONS

Pugs with a longstanding thoracolumbar myelopathy presented with a progressive clinical course and a high prevalence of fecal incontinence. The pathology, characterized by meningeal fibrosis with associated malacic spinal cord lesions, was accompanied by neighboring vertebral column lesions. In addition, lympho‐histiocytic CNS inflammation was recognized in the CNS related and unrelated to the focal spinal cord lesion. Increased knowledge of the clinical and pathological characteristics is important for our understanding and treatment of this debilitating condition in pugs. Future studies should focus on the mechanisms behind the development of meningeal fibrosis in pugs.

## CONFLICT OF INTEREST DECLARATION

Authors declare no conflict of interest.

## OFF‐LABEL ANTIMICROBIAL DECLARATION

Authors declare no off‐label use of antimicrobials.

## INSTITUTIONAL ANIMAL CARE AND USE COMMITTEE (IACUC) OR OTHER APPROVAL DECLARATION

Animal Ethics Committee of Sweden (Uppsala djurförsöksetiska nämnd) C202/2014.

## HUMAN ETHICS APPROVAL DECLARATION

Authors declare human ethics approval was not needed for this study.
